# P-107. Serious Injection-Related Infections in Hospitalized Adults with Opioid Use Disorder Enrolled in an Ongoing Randomized Clinical Trial: A Focus on *Staphylococcus aureus*

**DOI:** 10.1093/ofid/ofae631.314

**Published:** 2025-01-29

**Authors:** Laura Fanucchi, Alice C Thornton, Evelyn Villacorta Cari, Ryan P Mynatt, Connor VanMeter, Paul Nuzzo, Sharon Walsh, Loui Chang, Wyatt Kunzelman, Shashi Kapadia, Michelle Lofwall

**Affiliations:** University of Kentucky, Lexington, Kentucky; The University of Kentucky, Lexington, Kentucky; University of Kentucky, Lexington, Kentucky; University of Kentucky, Lexington, Kentucky; University of Kentucky, Lexington, Kentucky; University of Kentucky, Lexington, Kentucky; University of Kentucky, Lexington, Kentucky; University of Kentucky, Lexington, Kentucky; University of Kentucky, Lexington, Kentucky; Weill Cornell Medical Center, New York, New York; University of Kentucky, Lexington, Kentucky

## Abstract

**Background:**

*Staphylococcus aureus* (SA) causes the majority of serious injection-related infections (SIRI) in persons with opioid use disorder (OUD). Invasive SA infections are typically treated with prolonged IV antibiotic courses; increasingly oral transitions are considered though prospective clinical trial data are limited. Outpatient parenteral antibiotic therapy (OPAT) may be denied to persons with OUD and SIRI despite increasing evidence that it may be feasible and safe. Our ongoing study evaluates the efficacy and cost-effectiveness of an integrated outpatient care model combining Buprenorphine for OUD with OPAT (B-OPAT; NCT04677114) compared to Treatment As Usual (TAU) in persons hospitalized with OUD and SIRI. Here we present preliminary data on the prevalence of SA infections, treatment outcomes, and adverse events (AE) in the ongoing B-OPAT study.

**Figure:**
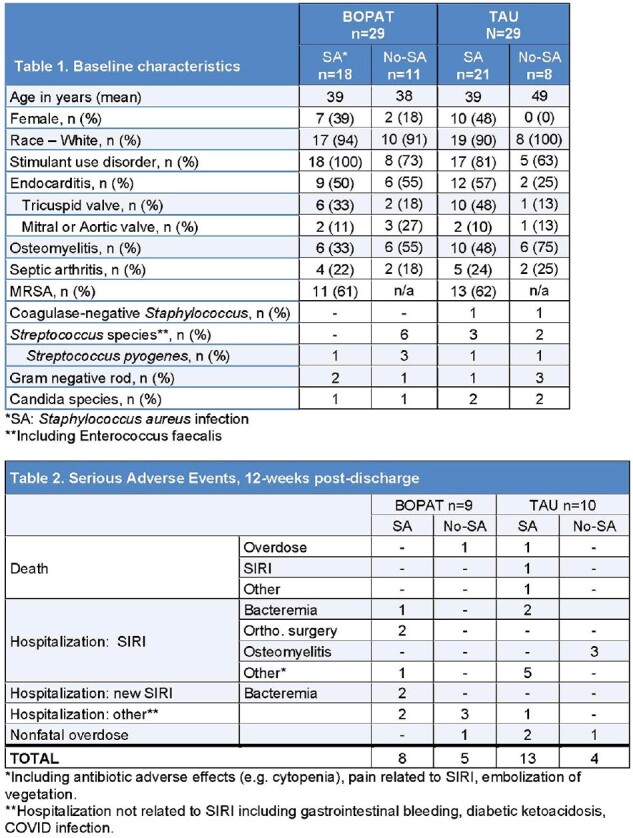

**Methods:**

This study is a randomized, 2-arm superiority trial enrolling hospitalized adults with OUD and SIRI. Participants are randomized 1:1 to discharge with B-OPAT or TAU. SIRI details, planned and completed days of IV antibiotic therapy, and AEs are collected at baseline and through the 12-week post-discharge intervention.

**Figure:**
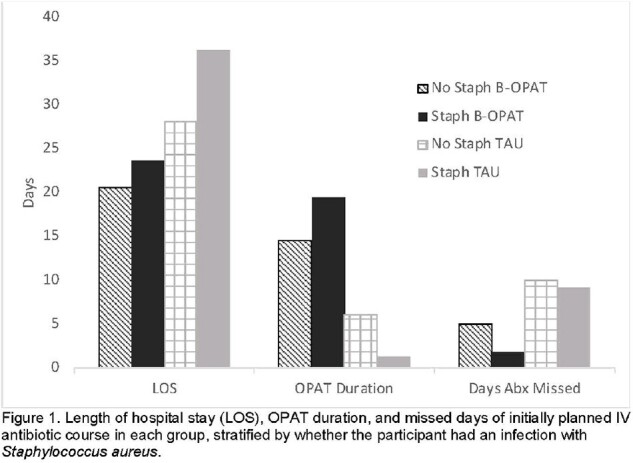

**Results:**

From March 2021 – March 2024, 58 persons were randomized (B-OPAT: 29; TAU: 29), with 39 (67%) having SA infections (B-OPAT: 18; TAU: 21), and of those 24 (61%) due to Methicillin-resistant SA (MRSA). Baseline characteristics stratified by SA are shown in Table 1. In patients with SA, average hospital length of stay was longer and fewer days of IV antibiotics were missed in both BOPAT and TAU (Figure 1). In BOPAT 1 person received oral antibiotic transition, and 8 in TAU, all with SA. Over 12-weeks post-discharge, B-OPAT had 13 serious adverse events (SAE) including 1 death in non-SA; 8 SAE were in those with SA. TAU had 17 SAE, 13 in those with SA including 3 deaths (Table 2).

**Conclusion:**

Our ongoing clinical trial enrolling hospitalized persons with OUD and SIRI shows that SA accounts for the majority of infections overall, and that SA may be associated with longer hospitalizations and higher morbidity. Importantly, our study may contribute prospective safety and other clinical trial data to inform the practice of oral transitions for invasive SA infections.

**Disclosures:**

**Sharon Walsh, PhD**, Astra Zeneca: Advisor/Consultant|Braeburn Pharmaceuticals: Advisor/Consultant|Cerevel Therapeutics: Advisor/Consultant|Lundbeck: Advisor/Consultant|Opiant Pharmaceuticals: Advisor/Consultant **Michelle Lofwall, MD**, Berkshire Biomedical: Advisor/Consultant|Braeburn Pharmaceuticals: Advisor/Consultant|Journey Colab: Advisor/Consultant

